# Association of rs662799 in APOA5 with CAD in Chinese Han population

**DOI:** 10.1186/s12872-017-0735-7

**Published:** 2018-01-08

**Authors:** Hua Chen, Shifang Ding, Mi Zhou, Xiayin Wu, Xi Liu, Yun Wu, Dechao Liu

**Affiliations:** 1 Department of Cardiology, Southern Medical University, Guangzhou, Guangdong China; 2 Department of Cardiology, Wuhan General Hospital of Guangzhou Military Region, NO.627, Wuyue Road, Wuhan City, Hubei Province China; 3 Department of Cardiology, Inner Mongolia People’s Hospital, Huhehaote, Inner Mongolia China; 4 Department of Cardiology, Inner Mongolia Medical University, Huhehaote, Inner Mongolia China

**Keywords:** Coronary artery disease, Chinese Han population, Case-control study, Single nucleotide polymorphisms

## Abstract

**Background:**

CAD (Coronary Artery Disease) is a complex disease that influenced by various environmental and genetic factors. Previous studies have found many single nucleotide polymorphisms (SNPs) associated with the risk of CAD occurrence. However, the results are inconsistent. In this study, we aim to investigate genetic etiology in Chinese Han population by analysis of 7 SNPs in lipid metabolism pathway that previously has been reported to be associated with CAD.

**Methods:**

A total of 631 samples were used in this study, including 435 CAD cases and 196 normal healthy controls. SNP genotyping were conducted via multiplex PCR amplifying followed by NGS (next-generation sequencing).

**Results:**

Rs662799 in APOA5 (Apolipoprotein A5) gene was associated with CAD in Chinese Han population (Odds-ratio = 1.374, *P*-value = 0.03). No significant association was observed between the rest of SNPs and CAD. Stratified association analysis revealed rs5882 was associated with CAD in non-hypertension group (Odds-ratio = 1.593, *P*-value = 0.023). Rs1800588 was associated with CAD in smoking group (Odds-ratio = 1.603, *P*-value = 0.035).

**Conclusion:**

The minor allele of rs662799 was the risk factor of CAD occurrences in Chinese Han population.

## Background

Coronary Artery Disease (CAD) is a common type of cardiovascular disease which is the leading cause of death worldwide [1]. In China, it was estimated that 700,000 people died from CAD each year [2]. Many environmental factors have been identified to be associated with CAD, including diabetes, hypertension, smoking, TG (Triglyceride), HDL-C (high-density lipoprotein cholesterol), LDL-C (low-density lipoprotein cholesterol), TC (Serum total cholesterol) [3]. Genetic variants also contribute to CAD risk [4, 5]. To date, Genome Wide Association Study (GWAS) studies have identified many SNPs to be associated with CAD among different populations [6–10], including SNPs located within or nearby lipid metabolism genes. Some of them were successfully replicated in different populations, but there are still inconsistent results in some researches.

In this study, we aim to investigate the association of 7 SNPs of lipid metabolism genes (LIPC rs1800588, LPL rs320, APOC3 rs5128, CETP rs5882, PON1 rs662, APOA5 rs662799, APOB rs693) with the risk of developing CAD in Chinese Han population.

## Methods

### Patients and controls

The participants in this study were recruited from Wuhan General Hospital of Guangzhou Military Region between 2010 and 2016. Individuals with incomplete information were excluded. A total of 435 CAD patients and 196 non-CAD controls were involved in the study. All the participants were unrelated Chinese Han individuals. This study was approved by the Medical Ethics Committee of Southern Medical University and compliant with the principles set forth by the Declaration of Helsinki Principles.

### Data collection

Subjects were defined as smokers if they smoked more than 100 cigarettes in lifetime. Subjects were diagnosed hypertension as systolic blood pressure (SBP) >140 mmHg or diastolic blood pressure (DBP) >90 mmHg [11]. Diabetes mellitus was defined as either fasting plasma glucose levels of ≥7.0 mmol/L or plasma glucose levels of ≥11.1 mmol/L [12]. Total cholesterol, HDL cholesterol, LDL cholesterol, C-reactive protein (CRP) and triglyceride (TG) levels were measured according to standard laboratory methods in Southern Medical University. The diagnostic criteria for CAD cases were defined as followings: at least one of the major segments of coronary arteries (right coronary artery, left circumflex, or left anterior descending arteries) with more than or equal to 50% organic stenosis based on coronary angiography.

### SNP genotyping

Blood samples (5 ml) were collected from participants. DNA extraction was conducted by TianGen DNA extraction kit (TianGen Ltd., Beijing, China) according to the manufacture instruction. DNA quality was analyzed by Electrophoresis and NanoDrop (NanoDrop Technologies, Houston, TX, USA) quantification. 20 ng DNA was used for PCR amplification. Chimeric specific primers were designed using Oligo 6.0, which contain target sequences and universal sequences. The product sizes of PCR reaction were between 107 and 160 bp, and the primer length was 37–38 bp, with melting temperature (Tm) 55–65 °C and the GC content between 20 and 80%. Multiplex PCR was conducted and followed by adaptor adding. Final products were purified by polyacrylamide gel electrophoresis (PAGE) and then sequenced on MiSeq platform (Illumina, USA).

### Sequencing data analysis

Sequencing data were separated by index sequences using FASTX-toolkit as each sample group has a unique index sequence. Then, the index and adapter sequences were trimed out with cutadapt. And target sequences were mapped to human genome reference sequence (NCBI, dbSNP bulid 142) using BWA software. SNPs were called by samtools.

### Statistical analysis

Continuous variables between cases and controls are calculated using Student’s t test and presented as mean ± SD. Categorical variables were calculated using chi-square test. Gene frequencies, allele frequencies, and differences in genotype and allele frequencies between different groups were also examined. Hardy-Weinberg equilibrium (HWE) test was performed by SHEsis [13]. *P* value >0.05 was considered in HWE. Allele distribution between cases and controls were analyzed using chi-square test. Odds ratios (ORs) and 95% confidence intervals (CIs) were calculated by logistic regression analysis after adjusting for age, hypertension, type 2 diabetes, TG, TC, HDL-C and LDL-C. *P* value <0.05 was considered as statistically significant. Association analysis were conducted by SPSS version 21.0 (SPSS Inc., Chicago, Illinois, USA).

## Results

### Clinical characteristics of cases and controls

In this study, 435 CAD patients and 196 controls were included. Clinical characteristics are summarized in Table 1. Samples in case group are more likely to have hypertension, diabetes and smoking. EF, HDLC and APOA1 levels are significantly lower in case group than in control group, while TG, APOA1, GLU and GHb levels are significantly higher in case group than in control group. These data indicate that male, EF, HDLC, TG, ApoA, APOA1, GLU, GHb, hypertension, diabetes and smoking are risk factors of developing CAD in this study.

**Table 1:** Baseline characteristics

	Cases	Controls	*P*
Participant (n)	435	196	
Gender (male, N (%))	144 (33.1%)	92 (46.9%)	0.001
Age (years)	60.8 ± 10.5	59.9 ± 10.4	0.322
EF	63.374 ± 8.058	64.757 ± 8.600	0.005
CRP	1.822 ± 2.891	1.137 ± 2.032	0.139
HDLC	1.116 ± 0.506	1.164 ± 0.303	0.006
LDLC	2.335 ± 0.778	2.242 ± 0.659	0.296
TC	4.379 ± 1.055	4.211 ± 0.896	0.102
TG	1.949 ± 1.835	1.5 ± 0.933	0.001
ApoA	222.737 ± 236.699	162.74 ± 218.465	0.001
APOA1	1.055 ± 0.213	1.155 ± 0.294	0.015
ApoB	0.784 ± 0.248	0.75 ± 0.203	0.609
GLU	6.046 ± 2.221	5.322 ± 1.375	0.001
GHb	5.924 ± 1.205	5.57 ± 0.903	0.007
Hypertension, yes, N (%)	282 (65.6%)	106 (54.1%)	0.006
Diabetes, yes, N (%)	123 (28.5%)	25 (12.8%)	0.000019
Smoker, yes, N (%)	206 (47.7%)	73 (37.6%)	0.019

### Association with the risk of developing CAD

Seven SNPs were genotyped in 435 cases and 196 controls. Allele frequency distribution was shown in Table 2. All SNPs were accorded with HWE. In these 7 SNPs, rs5882 and rs662799 were associated with CAD (*p* ≤ 0.05), in which rs662799 was explored by Ye et al. of the association between coronary heart disease (CHD) and the APOA5 rs662799 polymorphism [14]. After adjustment of age, gender, smoking, diabetes and hypertension, only rs662799 was significantly associated with increasing risk of developing CAD (risk allele T, OR = 1.374, 95% CI = 1.032–1.83, *P* = 0.03) (Table 3). Minor (T) allele frequency of SNP rs662799 in control and case group were 33.9 and 38.2% respectively. The results remained significant when HDLC, LDLC, TC and TG were introduced into the model (data not shown). No significant differences were observed in other polymorphisms after adjustment.

**Table 2:** Hardy-Weinberg equilibrium

SNP	Genotype	Cases				Controls			
		Actual	Expected	χ2	P	Actual	Expected	χ2	*P*
rs1800588	CC	149	147.1273	0.163509	0.685946	78	78.67222	0.050014	0.823039
	CT	178	181.7455			82	80.65556		
	TT	58	56.12727			20	20.67222		
rs320	GG	12	11.31992	0.059714	0.806948	7	4.666667	1.68	0.194924
	GT	107	108.3602			42	46.66667		
	TT	260	259.3199			119	116.6667		
rs5128	CC	44	46	0.195652	0.658253	21	16.8342	2.076069	0.149625
	CG	188	184			72	80.33161		
	GG	182	184			100	95.8342		
rs5882	AA	116	120.1608	0.681031	0.409232	74	68.04188	3.21016	0.073182
	AG	213	204.6784			80	91.91623		
	GG	83	87.1608			37	31.04188		
rs662	AA	51	50.9484	0.000125	0.991075	26	30.48262	1.854151	0.173301
	AG	186	186.1032			99	90.03476		
	GG	170	169.9484			62	66.48262		
rs662799	AA	211	206.1553	1.235773	0.266287	111	108.7513	0.759702	0.383422
	AG	170	179.6894			67	71.4974		
	GG	44	39.15529			14	11.7513		
rs693	CC	349	348.889	0.015305	0.901541	147	147.8421	0.974012	0.323682
	CT	35	35.22208			24	22.31579		
	TT	1	0.888961			0	0.842105

**Table 3:** association analysis

rs	Control	Case	P	OR	95%CI	FDR	Padj	OR	95%CI
rs1800588	0.661/0.339	0.618/0.382	0.163	1.205	0.927–1.566	0.228	0.104	1.257	0.954–1.656
rs320	0.833/0.167	0.827/0.173	0.803	1.045	0.741–1.472	0.803	0.963	0.991	0.693–1.418
rs5128	0.705/0.295	0.667/0.333	0.187	1.193	0.918–1.55	0.218	0.31	1.152	0.876–1.514
rs5882	0.597/0.403	0.536/0.464	0.048	1.281	1.002–1.638	0.168	0.128	1.222	0.944–1.581
rs662	0.596/0.404	0.646/0.354	0.098	0.809	0.629–1.04	0.229	0.123	0.813	0.624–1.058
rs662799	0.753/0.247	0.696/0.304	0.043	1.326	1.008–1.744	0.301	0.03	1.374	1.032–1.83
rs693	0.93/0.07	0.952/0.048	0.135	0.669	0.394–1.137	0.236	0.168	0.679	0.392–1.177

### Stratified association analysis

Gender, smoking, diabetes and hypertension status were stratified for further investigation (Table 4). Rs662799 was associated with CAD in male and hypertension group. Rs5882 was associated with CAD in non-hypertension group. Rs1800588 was associated with CAD in smoking group.

**Table 4:** Stratified analysis

	rs1800588			rs320			rs5128			rs5882			rs662			rs662799			rs693		
	Padj	OR	95% CI	Padj	OR	95% CI	Padj	OR	95% CI	Padj	OR	95% CI	Padj	OR	95% CI	Padj	OR	95% CI	Padj	OR	95% CI
Male	0.059	1.417	0.987–2.036	0.399	1.233	0.758–2.006	0.285	1.215	0.85–1.736	0.25	1.218	0.87–1.705	0.392	0.858	0.604–1.218	0.003	1.8	1.215–2.668	0.356	0.711	0.345–1.467
Female	0.835	1.047	0.68–1.61	0.294	0.747	0.433–1.288	0.742	1.075	0.7–1.651	0.32	1.227	0.819–1.839	0.146	0.739	0.492–1.111	0.936	0.982	0.637–1.514	0.316	0.646	0.275–1.519
Diabetes	0.961	0.982	0.472–2.042	0.447	1.484	0.536–4.106	0.686	1.154	0.576–2.309	0.105	1.738	0.891–3.394	0.591	0.831	0.422–1.635	0.226	1.576	0.754–3.291	0.091	0.323	0.087–1.197
No diabetes	0.073	1.313	0.975–1.767	0.695	0.926	0.629–1.363	0.374	1.145	0.849–1.543	0.362	1.14	0.86–1.51	0.155	0.811	0.608–1.082	0.067	1.34	0.98–1.832	0.392	0.77	0.423–1.401
Hypertension	0.206	1.269	0.877–1.835	0.313	1.28	0.792–2.069	0.158	1.297	0.903–1.863	0.929	0.985	0.7–1.385	0.429	0.869	0.613–1.232	0.004	1.792	1.199–2.679	0.233	0.615	0.277–1.366
No hypertension	0.24	1.285	0.846–1.954	0.214	0.7	0.399–1.228	0.969	0.992	0.649–1.516	0.023	1.593	1.067–2.379	0.14	0.736	0.49–1.106	0.992	1.002	0.657–1.529	0.515	0.775	0.36–1.668
Smoking	0.035	1.603	1.035–2.482	0.702	0.895	0.506–1.581	0.402	1.197	0.786–1.824	0.393	1.191	0.798–1.779	0.115	0.715	0.472–1.084	0.108	1.456	0.921–2.301	0.235	0.631	0.295–1.35
No smoking	0.625	1.094	0.763–1.57	0.811	1.058	0.667–1.677	0.511	1.13	0.785–1.626	0.273	1.209	0.861–1.699	0.451	0.876	0.621–1.236	0.123	1.339	0.924–1.941	0.552	0.783	0.35–1.753

## Discussion

CAD was a complex disease that influenced by a combination of genetic and environmental factors, but so far the molecular mechanisms of CAD were only partially revealed [15]. Lipoprotein metabolism was associated with CAD susceptibility in general population. LDLC, TC, TG, HDLC were associated with CAD status in many studies.

Many genes involving in lipid and lipoprotein metabolism pathway had been revealed to be related with CAD. Notably, SNPs of some genes that involved in lipid metabolism were identified to be associated with CAD developing risk. Many studies investigate the relationship between polymorphisms in or near these genes and CAD development. However, the results were inconsistent in different races or populations. The present study was performed to investigate if the polymorphisms in lipid metabolism genes were associated with CAD occurrences.

A significant association of the SNP rs662799 in APOA5 genes with CAD was observed after adjustment of gender, age, smoking, diabetes, hypertension and lipid status. However, no association was found between other SNPs and CAD susceptibility.

Apoa5 played an important role in TG metabolism (synthesis and removal of TG) [16]. TG was also a risk factor for CAD. APOA5 gene expression level was associated with TG plasma concentration [16]. Rs662799 was a polymorphism located in the promoter region (−1131 T > C) that influences the expression level of APOA5 gene [17]. The allele frequency of rs662799 in HapMap database was 1.7, 13.3. 26.7 and 28.9% in European, African, Chinese and Japanese respectively.

In previous researches, rs662799 was associated with TG level [18, 19]. In some studies, no association was detected between rs662799 and CAD [20], possibly due to lack of power.

There are limitations to the study. Our analysis only includes the study of Chinese Han population, and the sample size is small. So, it might not apply to other populations.

## Conclusion

In conclusion, we confirmed that rs662799 in APOA5 gene was significantly associated with CAD development. As the cohort size was limited, there may not be sufficient power to detect the effects of other SNPs. Besides of SNPs, rare mutations might also contribute to CAD development. Further studies with larger population and different technology were needed in order to provide more insights into the biological relevance of CAD.

## Abbreviations

APOA5: Apolipoprotein A5
CAD: Coronary Artery Disease
DBP: Diastolic blood pressure
GWAS: Genome Wide Association Study
HDL-C: High-density lipoprotein cholesterol
HWE: Hardy-Weinberg equilibrium
LDL-C: Low-density lipoprotein cholesterol
NGS: Next-generation Sequencing
PAGE: Polyacrylamide gel electrophoresis
SBP: Systolic blood pressure
SNPs: Single Nucleotide Polymorphisms
TC: Serum total cholesterol
TG: Triglyceride

## References

[1] Roger VL, Go AS, Lloyd-Jones DM, Adams RJ, Berry JD, Brown TM, Carnethon MR, Dai S, de Simone G, Ford ES (2011). Heart disease and stroke statistics--2011 update: a report from the American Heart Association. Circulation.

[2] Wang F, CQ X, He Q, Cai JP, Li XC, Wang D, Xiong X, Liao YH, Zeng QT, Yang YZ (2011). Genome-wide association identifies a susceptibility locus for coronary artery disease in the Chinese Han population. Nat Genet.

[3] Foody J, Huo Y, Ji L, Zhao D, Boyd D, Meng HJ, Shiff S, Hu D (2013). Unique and varied contributions of traditional CVD risk factors: a systematic literature review of CAD risk factors in China. Clin Med Insights Cardiol.

[4] Dai X, Wiernek S, Evans JP, Runge MS (2016). Genetics of coronary artery disease and myocardial infarction. World J Cardiol.

[5] McPherson R, Tybjaerg-Hansen A (2016). Genetics of coronary artery disease. Circ Res.

[6] Davies RW, Wells GA, Stewart AF, Erdmann J, Shah SH, Ferguson JF, Hall AS, Anand SS, Burnett MS, Epstein SE (2012). A genome-wide association study for coronary artery disease identifies a novel susceptibility locus in the major histocompatibility complex. Circ Cardiovasc Genetics.

[7] Dichgans M, Malik R, Konig IR, Rosand J, Clarke R, Gretarsdottir S, Thorleifsson G, Mitchell BD, Assimes TL, Levi C (2014). Shared genetic susceptibility to ischemic stroke and coronary artery disease: a genome-wide analysis of common variants. Stroke.

[8] Lu X, Wang L, Chen S, He L, Yang X, Shi Y, Cheng J, Zhang L, CC G, Huang J (2012). Genome-wide association study in Han Chinese identifies four new susceptibility loci for coronary artery disease. Nat Genet.

[9] Reilly MP, Li M, He J, Ferguson JF, Stylianou IM, Mehta NN, Burnett MS, Devaney JM, Knouff CW, Thompson JR (2011). Identification of ADAMTS7 as a novel locus for coronary atherosclerosis and association of ABO with myocardial infarction in the presence of coronary atherosclerosis: two genome-wide association studies. Lancet (London, England).

[10] Samani NJ, Erdmann J, Hall AS, Hengstenberg C, Mangino M, Mayer B, Dixon RJ, Meitinger T, Braund P, Wichmann HE (2007). Genomewide association analysis of coronary artery disease. N Engl J Med.

[11] Mancia G, Fagard R (2013). Guidelines for the management of hypertension and target organ damage: reply. J Hypertens.

[12] Giuseppe M, Robert F (2013). Guidelines for the management of hypertension and target organ damage. J Hypertens.

[13] Shi YY, He L (2005). SHEsis, a powerful software platform for analyses of linkage disequilibrium, haplotype construction, and genetic association at polymorphism loci. Cell Res.

[14] Ye H, Zhou A, Hong Q, Tang L, Xu X, Xin Y, Jiang D, Dai D, Li Y, Wang DW (2015). Positive association between APOA5 rs662799 polymorphism and coronary heart disease: a case-control study and meta-analysis. PLoS One.

[15] Iannaccone M, Quadri G, Taha S, D'Ascenzo F, Montefusco A, Omede P, Jang IK, Niccoli G, Souteyrand G, Yundai C (2016). Prevalence and predictors of culprit plaque rupture at OCT in patients with coronary artery disease: a meta-analysis. Eur Heart J Cardiovascr Imaging.

[16] De Andrade FM, Maluf SW, Schuch JB, Voigt F, Barros AC, Lucatelli JF, Hutz MH (2011). The influence of the S19W SNP of the APOA5 gene on triglyceride levels in southern Brazil: interactions with the APOE gene, sex and menopause status. Nutr Metab Cardiovasc Dis.

[17] Pennacchio LA, Olivier M, Hubacek JA, Cohen JC, Cox DR, Fruchart JC, Krauss RM, Rubin EM (2001). An apolipoprotein influencing triglycerides in humans and mice revealed by comparative sequencing. Science (New York, NY).

[18] Ramakrishnan L, Sachdev HS, Sharma M, Abraham R, Prakash S, Gupta D, Singh Y, Bhaskar S, Sinha S, Chandak GR (2011). Relationship of APOA5, PPARgamma and HL gene variants with serial changes in childhood body mass index and coronary artery disease risk factors in young adulthood. Lipids Health Dis.

[19] Takeuchi F, Isono M, Katsuya T, Yokota M, Yamamoto K, Nabika T, Shimokawa K, Nakashima E, Sugiyama T, Rakugi H (2012). Association of genetic variants influencing lipid levels with coronary artery disease in Japanese individuals. PLoS One.

[20] Zhou J, Xu L, Huang RS, Huang Y, Le Y, Jiang D, Yang X, Xu W, Huang X, Dong C (2013). Apolipoprotein A5 gene variants and the risk of coronary heart disease: a casecontrol study and metaanalysis. Mol Med Rep.

